# *Drosophila* video-assisted activity monitor (DrosoVAM): a versatile method for behaviour monitoring

**DOI:** 10.1098/rsos.250764

**Published:** 2025-09-04

**Authors:** Maxime Revel, Emi Nagoshi, Robert Maeda

**Affiliations:** ^1^ Department of Genetics and Development, University of Geneva, Geneva, Switzerland

**Keywords:** behaviour, *Drosophila*, circadian rhythm, method

## Abstract

*Drosophila melanogaster* has been a pioneering model system for investigations into the genetic bases of behaviour. Studies of circadian activity were some of the first behaviours investigated in flies. The *Drosophila* Activity Monitoring (DAM) system by TriKinetics played a key role in establishing the fundamental feedback loop of the circadian clock. Although this method has many times proven to be extremely useful, it suffers from its simplification of activity to the interruption of an infrared (IR) beam. It is blind to fly movements not disrupting the beam and any modifications to this assay to achieve better resolution often requires the purchase of new and expensive modules. We required a relatively high-throughput system to explore the potential post-mating activity changes of larger *Drosophila* species. Rather than investing in a larger and more complex DAM system, we designed a new monitoring system that is more versatile, economic and sensitive than DAM. This new system, called DrosoVAM (*Drosophila* Video-assisted Activity Monitoring), is simple to implement and cost efficient, using a Raspberry Pi-controlled IR, digital video system to record multiple chambers and Python scripts that drive the deep learning software DeepLabCut, to track fly activity over multiple days.

## 1. Introduction

Model organisms continue to play a critical role in deciphering the links between neuronal circuits and complex behaviours [1–5]. In particular, *Drosophila melanogaster* has helped in unravelling the cellular and molecular basis of complex behaviours like memory formation [6,7], aggression [8,9], sleep [10] and courtship [11–13]. Recently, the fruit fly has even proven to be useful in modelling behaviours found in human neurodegenerative diseases such as Alzheimer’s disease [14] and Parkinson’s disease [15].

As our understanding of *Drosophila* behaviour grows, we are becoming increasingly aware of the limitations of some of the standardized assays. One frequently used example of this is the TriKinetics *Drosophila* Activity Monitoring (DAM) system [16] used to monitor locomotor activity. This method places individual flies into narrow tubes with a minimal food source (5% sucrose, 2% agar) located at one end. Multiple tubes are then inserted into a monitor that emits an infrared (IR) beam through the centre of each tube and records the number of times the fly disrupts the IR beam. Due to its simplicity of use and high-throughput nature, this system has proven to be extremely useful. However, its spatial resolution is low, as the fly movements are generally monitored only in the centre of the tube. Furthermore, the restricted nature of the chambers limits the movements of the fly in unnatural ways.

Due to the limitations of methods like DAM, different approaches are being developed to track the behaviour of individual flies more precisely [17] (for a general comparison of these systems, see electronic supplementary material, table S1). Some of these systems use deep-learning algorithms to track, define and quantify micromovements such as proboscis extension or repositioning of halteres and antennae [18]. Other software and protocols are more specifically designed to identify behaviours of interest, such as egg laying [19] or feeding [20]. In contrast to DAM, many of these methods surprisingly suffer from being of too high a resolution [19–21], making data analysis more difficult and thereby reducing the number of individuals analysed per experiment.

In our laboratory, we were interested in using a high-throughput system like DAM to examine potential post-mating changes in the activity of other *Drosophila* species like *D. virilis* and *D. hydei*. However, initial trials using the DAM system highlighted some of its limitations. First, the tubes used for the DAM system present in our laboratory (designed for *D. melanogaster*) were too narrow to support the free movement of larger species like *D. virilis* and *D. hydei*. Second, the minimal food source used in DAM assays is known to be insufficient to support normal egg laying of females [22] and our attempts to replace the minimal food with standard, rich food proved to be problematic due to its propensity to dry out in such a small volume and the obscuring influence of hatching larvae over time.

To circumvent these difficulties, we designed a new monitoring system that is simple, more versatile, economic and sensitive than the DAM monitoring technique. Dubbed DrosoVAM for *Drosophila* Video-assisted Activity Monitor, this system is made up of three parts. The first part consists of an IR digital video system that records flies in 3D-printed chambers illuminated by an IR light source. As the circadian clock of *Drosophila* is not altered by IR light [23], IR monitoring can be used to track flies during both day and night cycles (as imposed by changing visible light) with equal sensitivity. In the second part, the digital recordings are cropped and then analysed by the deep learning software DeepLabCut [24,25] to track the flies in the chambers and convert the videos to positional values. Finally, in the third step, a set of Python scripts are run to interpret the tracking data and infer the movement behaviours of the flies. The versatility of the Python script, as well as the ease of 3D printing, makes the method highly flexible and inexpensive to modify in order to test for activity in a variety of situations. Here, we demonstrate the utility of this method in basic activity monitoring as well as in a post-mating food preference assay.

## 2. Material and methods

**Figure 1.**
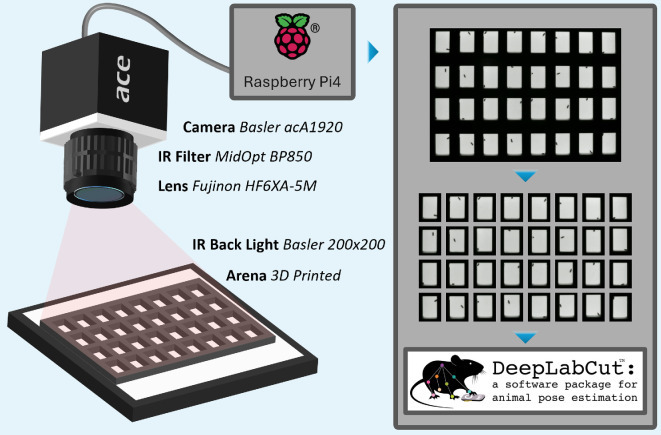
Hardware used to monitor flies using DrosoVAM and overview of the video analysis. Flies are placed in a chamber laying horizontally on an IR backlight and are recorded from above using a camera equipped with an IR pass filter and lens. The camera is connected to a Raspberry Pi4 on which a custom script is run to capture videos, here at 5 fps to limit file size. Videos are then cropped into individual videos each focused on one chamber and analysed using the tracking software DeepLabCut.

The DrosoVAM system (figure 1) is designed as a three-step protocol, each step of which is described here. All software used, STL files for 3D printing, along with the hardware list, are openly available and can be found on the associated GitHub repository (https://github.com/LabMaeda/DrosoVAM/).

### 2.1. Hardware—video acquisition and preparation

To ensure maximum versatility and ease of troubleshooting, we developed our own recording arena, 3D-printed in polylactic acid using a Prusa Mk3 filament printer. The recording arena measures 196 mm × 145 mm and is enclosed between two removable, 2 mm thick acrylic sheets (purchased through various suppliers on amazon.fr). The locomotor activity arena (electronic supplementary material, figure S1a) is composed of 32 chambers (23 mm × 15 mm × 5 mm), each with a small opening (3 mm diameter), through which the fly can be inserted, and a 5 mm wall at the ‘bottom’ to help contain the food while pouring. Alternatively, a food preference arena (electronic supplementary material, figure S1b) was designed with 24 chambers (50 mm × 10 mm × 5 mm) that have two walls to support food at each extremity.

The arena, once filled with standard cornmeal food and loaded with flies, was placed horizontally on top of an IR light source (Basler IR Back Light 200 × 200). Recording was performed using a 1920 p × 1080 p camera (Basler ac1920-25um) equipped with a lens (Fujinon HF6XA-5M) and an IR-pass filter (MidOpt BP850) (figure 1). The filter used on the camera lens completely blocked any non-IR light, so images recorded during either light or dark phases are identical, and tracking is not impaired by shifting light and dark phases.

The camera was connected to a Raspberry Pi 5 running a custom Python script that uses the pypylon wrapper from Basler AG to produce series of 2 h long video segments at 5 fps. This relatively low frequency allowed the videos to not exceed 400 MB, thus avoiding working with large files, all while keeping enough time resolution to monitor fly movements in the chambers. Although the recording was performed using a Raspberry Pi computer, more powerful machines could also be used if they are able to run Python scripts and to store the videos.

Videos were then cropped into smaller videos, each focusing on one chamber, using a second Python script that uses ffmpeg-python. This script works by defining the arena in a graphical interface to divide the whole viewing frame into a defined number of columns and rows based on the arena being used.

### 2.2. Software I—tracking

To optimize the tracking of the flies in the chambers, we took advantage of DeepLabCut software [24,25] which allows for marker-less tracking of various model animals. The string of 2 h videos of each chamber was loaded into the software and analysed using a trained network to identify flies at our level of resolution. The network we used, called ‘DrosoVAM_V3’, has been trained from a ResNet-50 network for 750 000 iterations using around 1000 labelled frames extracted from several videos. Only frames where a fly’s position was properly identifiable were labelled to avoid the introduction of mislabelled data in the training.

Analyses were performed using a workstation equipped with a GPU (NVIDIA RTX-4000 Ada generation), using DeepLabCut v. 2.3.9, Python v. 3.10.11, TensorFlow v. 2.10, CUDA v. 11.8 and CudNN v. 8.9.7.

### 2.3. Software II—behaviour analysis and interpretation

To transcribe tracking data into behaviours that are interpretable, we developed Python scripts to read the .csv files produced by DeepLabCut, analyse them and generate plots corresponding to the information in which we were interested. To maximize the number of flies that can be monitored simultaneously, we decided to use a camera that records the chamber from a distance of around 25 cm. In our experiments, this allowed for a reasonable trade-off between the number of chambers examined and tracking precision. While misdetection by DeepLabCut was rare, it occasionally happened. To help control for this, we included a step in our analysis that: (1) identifies the limits of the chamber; (2) identifies the frames where the flies are detected outside of the chamber; and then (3) replaces the coordinates by the average position between the values of the preceding and next detections located inside the chamber.

For each fly, frame-to-frame distances were summed for the bin durations. For each 10, 30 or 60 min bin, the average distance was calculated. Similarly, in the virtual DAM, the number of detections along the midline of the chambers was summed for the bin durations, and the median of all flies was calculated for visualization. Position analyses were carried out in a similar manner, with the position of the flies being first averaged for each second, then averaged over the bin duration. The average position of all flies of a given mating status was then calculated for visualization. For food preference assays, the frequency of the fly localization was calculated for each fly and for each spatial bin, counting for how many frames the fly was located in each area of the chamber.

To increase the accuracy of our analysis, we decided to focus on the average activity of a pool of flies each time. For locomotion activity monitoring, this pool was of 16 flies, while it was of 12 flies for the food preference assays. While the data for each individual are available, the general noisiness of them makes it hard to use in comparison analysis (electronic supplementary materials, figures S2 and S3).

### 2.4. *Drosophila* activity monitoring assay

DAM assays were carried out as described in Dorcikova *et al.* [26] with minor modifications due to the food composition. After mating completion, flies were placed in individual glass tubes containing standard cornmeal food to allow mated females to lay eggs. Tubes were placed in the DAM System 3 (Trikinetics). Locomotor activity data were recorded for 3–9 days. The extracted sleep quantity from the activity monitors was treated in 1, 10, 30 or 60 min bins using a Python script for data visualization.

## 3. Results

**Figure 2.**
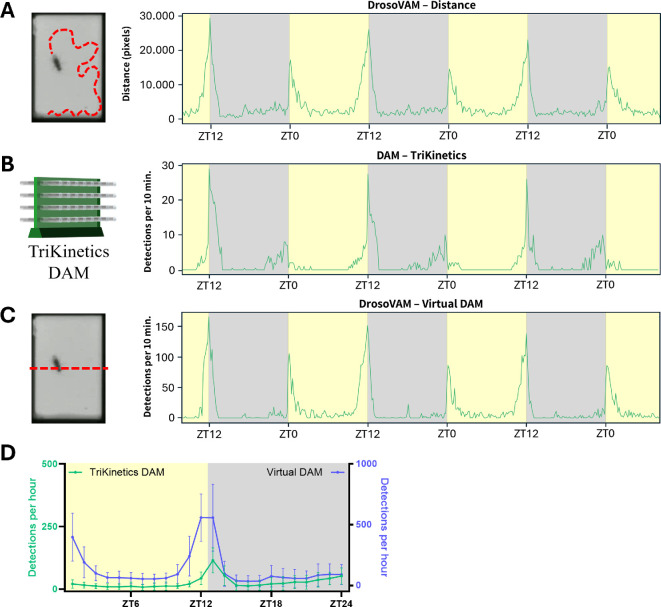
Locomotor activity of virgin *D. melanogaster*. Light/dark (L/D) periods are shown with yellow/grey background, respectively. (A) Average distance (pixels) moved by the flies (*n* = 16) estimated using DrosoVAM. (B) Activity monitored using the TriKinetics DAM system (*n* = 32). Median number of detections per bin of 10 min. (C) Virtual DAM analysis simulated from DrosoVAM videos (*n* = 16). Median number of detections per bin of 10 min. (D) Comparison of average day activity, measured with DAM (green, left *y*-axis) or DrosoVAM virtual DAM (blue, right *y*-axis). Average day calculated from 2 full days of monitoring, binning of 60 min.

Due to our interest in monitoring the female post-mating changes in activity in flies of different species, we sought an alternative to the traditional DAM system. For this, we developed a simple video recording-based system that we call DrosoVAM (detailed in figure 1). To confirm that DrosoVAM can track activity, we first looked at virgin female *D. melanogaster* and assessed the capacity of DrosoVAM to replicate results obtained with DAM. In these experiments, we analysed the distance covered by each virgin female in 10 min bins, as measured by our DrosoVAM protocol (figure 2A) and compared it with the number of crossings detected per 10 min by the DAM system (figure 2B). Overall, DrosoVAM largely replicates the activity pattern derived from the DAM system; the periodicity of the activity peaks surrounding the light changes is distinctly visible, particularly, the evening activity increase (figure 2A,B; for representative individual traces, see electronic supplementary material, figure S2). However, subtle differences can also be seen. As the measurements used by the two methods are of different natures (number of detections versus distance moved), direct statistical comparison is impossible. We attempted to bypass this by mimicking the DAM assay using our DrosoVAM videos. We did this by simulating a virtual laser in the middle of the chamber and counting how many times the fly crosses this virtual line (figure 2C). We then averaged several days of monitoring to compare the results of DAM with our ‘virtual DAM’ assay (figure 2D). Comparing the two experiments, we find that the general pattern of activity seems to be similar, but that the daytime activity was significantly higher when monitored using the DrosoVAM system. Furthermore, both the morning peak, triggered by light onset, and the anticipatory evening peak were more pronounced in the DrosoVAM system.

**Figure 3.**
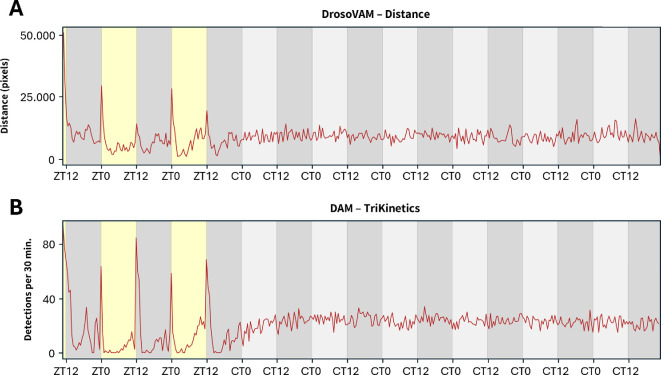
Locomotor activity of male *per^01^ D. melanogaster* recorded using the DrosoVAM and the DAM systems. Light and dark periods are shown in yellow and grey background, respectively. In constant darkness, subjective day is shown in light grey. 30 min bins, 204 h of monitoring. (A) Average distance (in pixels) moved by the flies (*n* = 16) estimated using DrosoVAM. (B) Activity monitored using the TriKinetics DAM system (*n* = 32). Median number of detections per bin of 30 min.

These initial results encouraged us also to examine flies in which circadian activity is disturbed. We turned to *per* null mutant flies (*per^01^*) that lack the endogenous molecular clock [27]. Under LD (light–dark) cycles, *per^01^* flies display distinct startle activity peaks at the lights-on and lights-off transitions and become arrhythmic in constant darkness (DD, dark–dark) [28]. Consistent with these previous findings, locomotor activity of *per^01^* flies recorded using DrosoVAM lost all rhythmicity when switching from LD to DD (figure 3A) (see also electronic supplementary material, figure S3, for representative individual traces and a comparison with *w1118* control flies), in a fashion similar to our recordings using the DAM system (figure 3B). Aside from this observation, we also confirmed the capacity of our system to be used for long-running experiments (here for 8 days). In our hands, we have found out that experiments can be run for up to 10 days before the food becomes too dry for flies to survive.

**Figure 4.**
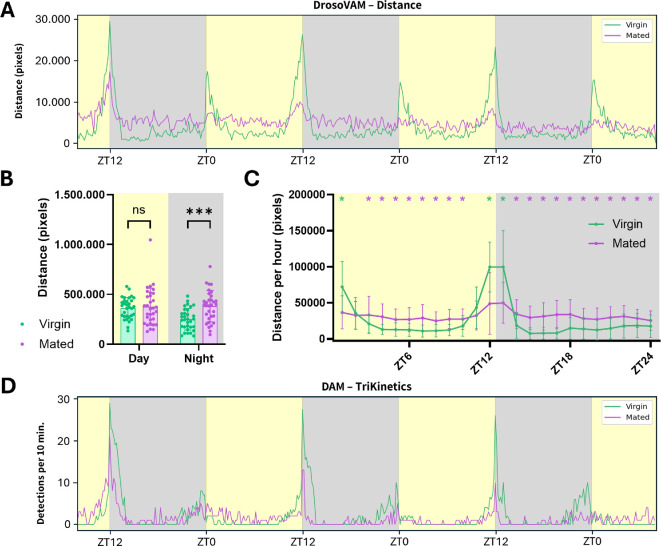
Activity comparison between *D. melanogaster* virgin and mated females. Virgins are shown in green, mated females in purple. L/D periods are shown with yellow/grey backgrounds, respectively, in 10 min bins over 72 h of monitoring. (A) Average distance (in pixels) moved by the flies (mated *n* = 16/virgin *n* = 16) estimated using DrosoVAM. (B) Total distance moved by flies during complete day and night periods (12 h/12 h). Mating status is compared for both periods using *t*‐test (day: *p* = 0.7129; night: *p* = 0.0002). (C) Comparison of the average activity measured using DrosoVAM distance analysis on virgin and mated females over a 24 h period. The average day was calculated from 2 full days of monitoring, with bins of 60 min. The impact of mating status has been tested for each hour using multiple Mann–Whitney tests (no star—no statistical discovery; one star—discovery; the colour of the star—which one of the conditions is higher). (D) Activity analysis using the TriKinetics DAM system (*n* = 32/32). Median number of detections per bin of 10 min.

In a first attempt at identifying behavioural post-mating responses using DrosoVAM, we placed freshly mated *D. melanogaster* females into monitoring chambers for video recording over the subsequent 3.5 days under a LD cycle (figure 4A). Using this assay, we find that mated females are significantly more active during the night periods relative to virgin flies (figure 4B). As DrosoVAM works through video, we were able to visually confirm that these changes in activity were real and not an artefact of our tracking method. On closer examination, mated flies seem to show lower activity peaks at the L/D transitions (figure 4C) combined with a reduction of the midday siesta and nighttime sleep behaviours that characterize the circadian behaviour of virgin females (figure 4C). Using DAM, we also see a reduction in the L/D transition activity peaks (figure 4D), but we do not see the overall activity changes that we see with DrosoVAM.

**Figure 5.**
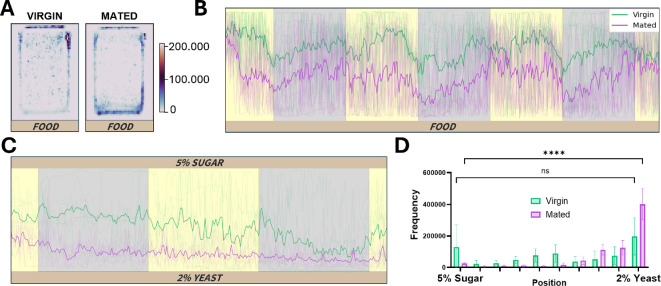
Position analysis of virgin and mated *D. melanogaster*. Virgin flies are shown in green and mated flies are shown in purple. Light and dark periods are shown with the yellow and grey background, respectively. (A) Heatmaps showing the position of the flies in the chambers over a 72 h period. The side of the chamber where the food is located is represented at the bottom of the heatmap. (B) Temporal representation of the position of the flies in the locomotor activity chambers. The extremity of the chamber in which food is present is located at the bottom of the chart (as indicated). Positions of all the flies are shown as thin lines, and the average position is represented with the thicker line. (C) Temporal representation of the position of the flies in the food preference chambers. Sugar food is located at the top of the chart (indicated as 5% sugar) and yeast is at the bottom (indicated as 2% yeast). (D) Histogram of the position of the flies along the food preference chambers. The chambers are divided into 10 bins (~5 mm per bin) and *t*‐tests have been performed between the bins closest to the foods.

We wondered what could account for the differences between these two systems in both virgin and mated flies. Because our system continuously records the fly position, we mapped the fly position, overall and over time. From this analysis, we find that mated flies tend to spend most of their time moving near/on the food while virgin flies tend to stay away from the food (figure 5A,B). This greater proximity to the food could be explained by the need of mated females to lay their eggs on the food, along with the necessity for higher nutrient intake after mating. As smaller movement near the food would not be recorded by DAM, this behaviour would go largely unnoticed. Furthermore, previous results using DAM have shown that virgin females show a higher activity in the late hours of the night relative to mated females [29]. We also observe this in the hours just prior to experimental dawn using DAM (figure 4B). However, using DrosoVAM, this period of activity is much less apparent, probably due to the generally higher level of nighttime activity observed.

As mated females seemed to spend more time near the food, we wanted to test if DrosoVAM could be adapted to study feeding behaviours in mated females. For this, we have developed an alternative version of the monitoring chamber (electronic supplementary material, figure S1b) that can be filled with two different types of food, allowing us to test for a possible shift in food preference. In this test, we put mated and virgin flies into chambers with 1% agar supplemented either with 2% yeast extract (source of proteins) or with 5% sucrose (source of carbohydrates). Previously, it was shown that *D. melanogaster* females change their food preference towards high-protein sources after mating [30,31]. Demonstrating the versatility of our system, we can confirm that mated flies spent more time near the yeast-containing food while virgin flies show little or no preference (figure 5C,D).

## 4. Discussion

The TriKinetics DAM system has made it possible to quantify the differences in behaviour between different *D. melanogaster* genotypes under different conditions [16,32]. The system has been highly adopted due to its simplicity and its scalability to large numbers. However, its simplification of activity to crossing an IR beam at the centre of a tube may lead to problems in interpreting the phenotypes being scored; movements made away from the centre line are lost, while small movements near the beam can give a false sense of hyperactivity. Modern video monitoring technology has now made using such a proxy for activity unnecessary for many applications. Here, we have shown that a video-based system like DrosoVAM is able to provide comparable, if not more complete results relative to DAM for the monitoring of *Drosophila* displacement activity. For example, previous DAM experiments have shown that mated females are more active than virgin flies during the day and described it as a post-mating reduction in siesta sleep [32]. Here, using DrosoVAM, we show that mated females actually display an almost constant level of activity that is higher than that of virgin females during periods of time in the day and night phases (figure 4A). Thus, the reduction in daytime siesta sleep seen in DAM, while real, is an incomplete picture of the activity changes experienced by mated females.

We can propose two likely reasons that the DAM system does not find a nighttime increase in activity. The first possibility is that the discrepancy between DAM and DrosoVAM results stems from a change in the localization of the flies that is not seen by DAM, while the second suggests that the change in behaviour is linked to the difference in environment between the two systems.

Regarding the first possibility, previous work has shown that isolated females prefer to lay their eggs in the dark [33]. Thus, it seems likely that mated females would spend more time closer to the food at night to lay eggs. Our DrosoVAM results are consistent with this interpretation. While mated females are more active than virgin flies (particularly during the nighttime hours; figure 5B), they tend to spend more of their time at or near the food than virgin flies. Because of this relocation, females could be more active at night, but this activity would be less likely to activate a DAM activity sensor located at the tube midline. If this interpretation is correct, it highlights an important caveat in using DAM or any system that proxies the frequency of a specific event to measure another. In many articles, periods of DAM inactivity (5 consecutive minutes without the fly crossing the central beam) have been labelled as sleep. Our work has shown that mated flies are often active on or near the food (figure 5A,B), potentially leading to falsely attributing egg laying or feeding behaviours as sleep when activity is not directly monitored. While we do not discount a potential inverse proportionality of the beam-crossing behaviour to sleep activity, particularly in individual males or virgin females as seen in [26,34–36], our DrosoVAM experiments show that potentially unrelated changes in behaviour might cause a quantitative misattribution of sleep bouts in flies.

An alternative possibility to explain the differences between the DAM and DrosoVAM results in overall activity is that flies behave differently in the confined environment of tubes relative to the more open environment of our chambers. Indeed, in the context of DAM, the animals are only given a few millimetres of space in which they can move in two of the three axes. As the size and characteristics of the chambers in which the flies are observed have been shown to affect their behaviour, this is a relevant concern [37–39]. We chose the size of our DrosoVAM chambers to allow for the monitoring of multiple flies, while providing each fly with an ample supply of food and a space sufficient for pivoting and movement in all directions. Furthermore, while a wider environment may explain the discrepancies in activity levels, it is possible that it would also affect the activity pattern itself. Indeed, in our DrosoVAM experiment on virgin females, aside from the startle activity response to light transitions, we failed to identify the stereotypical ‘morning anticipation’, previously characterized using narrower chambers. Additionally, studies using the DAM system have found that the richness of the food may alter the sleeping behaviour of mated females [40]. However, we ruled out any impact of food composition in the discrepancy we characterized, as the food used for both DAM and DrosoVAM experiments was identical.

Given the almost infinite number of variables that one can test when studying behaviour, one of the strengths of DrosoVAM is that it can be easily and inexpensively modified to fit one’s needs. For example, as a follow-up to our mated female localization results, we created a second setup with two different feeding areas per chamber to monitor where mated females would choose to spend their time. Using this assay, we were able to highlight how mated females display a striking preference for rich food (2% yeast) that is not seen with virgin flies (figure 5C,D). This result is consistent with previous studies using alternative methods (food dyes) [30,31], and shows how DrosoVAM can be quickly and easily modified to monitor different aspects of behaviour without the need to invest in more expensive equipment. The subsequent analysis of DrosoVAM data can also easily be adapted *a posteriori*, for example, to quantify sleep based on locomotor activity. While the quantification of sleep is theoretically possible, we decided to focus primarily on raw locomotor activity to assess changes in the behaviour of mated females, avoiding the misattribution of sleep to immobile flies that could be eating or laying eggs. Furthermore, despite the focus of our analysis on average data from 12 to 16 flies, we believe that it is possible to extract information also from individual data. For example, in our constant darkness experiment, we could verify that the average flatline activity observed was not resulting from asynchronous rhythm shifts, but rather that all *period* flies started to move constantly. Nevertheless, the noisiness of the individual data suggests that pooling individuals still is a better approach to locomotion monitoring. Finally, we have also been able to use DrosoVAM with other species of *Drosophila* like the larger flies *D. virilis* and *D. hydei*. Preliminary results using these species with DrosoVAM actually show that the system not only works but also functions better due to the larger size and darker IR profiles of these flies. Thus, the versatility of DrosoVAM in experiment design is not limited to modification of chambers and types of analysis, but also to the species that can be used in it.

Not only can DrosoVAM easily be implemented and modified but also it is noticeably easier to set up an experiment compared to the DAM system. Indeed, the preparation of the chamber for monitoring can take as few as 5 min, the time necessary to pour food into the food wells. The placement of the flies in the chambers can take place as soon as the food is solid, and takes a limited amount of time. Altogether, setting up the experiment and starting the recording takes less than an hour, allowing for the rapid start of monitoring.

Given the limitations of the DAM system, it is not surprising that several video-based monitoring systems have been developed by other groups. Many of these systems have been specifically designed to monitor more detailed behaviours, using higher resolution cameras over shorter periods of time or with fewer flies to keep the data manageable [18–21,41]. Still, other systems may be able to perform similar functions and at similar scales to DrosoVAM [37,42–47]. For comparison of some existing methods, see electronic supplmentary material, table S1. Our choice to develop DrosoVAM was driven, not because of the lack of other systems, but because adapting these systems to our varying needs proved to be harder than developing an inexpensive, simpler, modular system using easily obtainable parts and software. In conclusion, most components of the DrosoVAM pipeline are openly available through the associated GitHub repository (https://github.com/LabMaeda/DrosoVAM), similarly to DeepLabCut which can be found on its own repository. Thus, DrosoVAM’s strength lies in its providing an accessible, reliable and robust method to monitor displacement activity that is simple both to implement and to evolve as one sees fit.

## Data Availability

The .stl files for the 3D printing of the chambers and all Python scripts are available on the associated GitHub repository (https://github.com/LabMaeda/DrosoVAM/). All the data used to generate the graph presented here can be found on the associated Dryad repository [48]. Electronic supplementary material is available online [49].
